# Hydatid Cyst Rupture Due to Albendazole Treatment

**DOI:** 10.1590/0037-8682-0153-2025

**Published:** 2025-07-07

**Authors:** Gökhan Polat, Büşra Şahin Toprak, Enes Yılmaz

**Affiliations:** 1 Department of Radiology, Medical Faculty, Kutahya Health Sciences University, Kutahya, Turkey.

**FIGURE 1:**
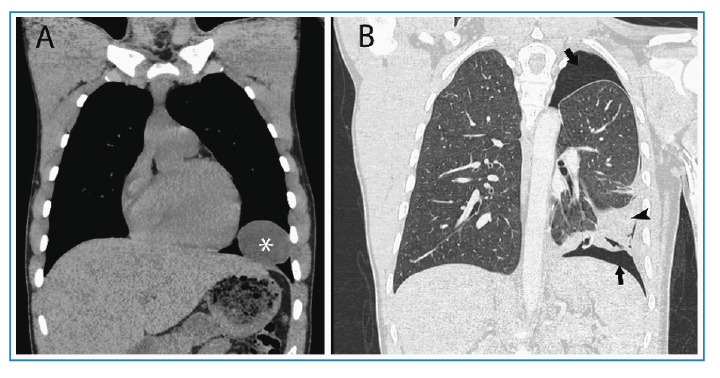
Non-contrast coronal thoracic computed tomography **(A)** showing a well-defined cystic lesion in the lower lobe of the left lung in the mediastinal window (asterisk). The cyst density measured to be 10 HU. Contrast-enhanced coronal thoracic computed tomography **(B)** demonstrating air densities consistent with pneumothorax between the pleural layers in the parenchymal window (black arrows). Additionally, marked wall irregularity and pleuropulmonary fibroatelectatic changes with volume loss are observed at the previously identified location of the cyst in the lower lobe of the left lung (black arrowhead).

A 17-year-old male patient presented to our hospital four months ago with complaints of cough, difficulty in breathing, and a stabbing sensation. A non-contrast thoracic computed tomography (CT) scan revealed a well-defined cystic lesion in the lower lobe of the left lung (Figure 1A). Oral albendazole was initiated for hydatid cyst (HC) treatment. In the fourth month of treatment, the patient presented with hemoptysis and dyspnea. A posteroanterior chest radiograph revealed extensive pneumothorax necessitating chest tube placement. A thoracic CT scan confirmed rupture of the cyst in the left lung (Figure 1B).

The hydatid disease belongs to the *Echinococcus* species that primarily affects the liver and lungs. HC progresses slowly and can remain asymptomatic. Pulmonary HC typically become inactive due to fibrosis 5-9 months after treatment^1^. Albendazole has been used to treat HC for approximately five decades. Additionally, they facilitate cyst treatment by activating certain cellular death mechanisms in the germinative membrane^2^. Some studies have reported ruptures as a complication of albendazole therapy. Notably, the study by Kürkçüoğlu et al.^2^ provides examples of such cases. Although these compounds are effective therapeutic agents for hydatid cysts, they are associated with a non-negligible risk of complications.
